# Creatinine and cystatin C-based indices for predicting sarcopenia, frailty and disability in older community-dwelling adults

**DOI:** 10.1016/j.jnha.2025.100635

**Published:** 2025-07-24

**Authors:** Anying Bai, Juan Xu, Weihao Xu, Jian Cao, Bei Zhao

**Affiliations:** a Department of Epidemiology and Biostatistics, School of Population Medicine and Public Health, Chinese Academy of Medical Sciences and Peking Union Medical College, Beijing, 100730, China; b Department of General Surgery, Affiliated Xiaoshan Hospital, Hangzhou Normal University, Hangzhou, 311231, China; c Department of Geriatrics, Guangdong Provincial Geriatrics Institute, Guangdong Provincial People's Hospital, Guangdong Academy of Medical Sciences, Southern Medical University, Guangzhou, 510080, China; d The Forth Healthcare Department of the Second Medical Center, Chinese PLA General Hospital, Beijing, China; e Department of Cardiology, The Ninth Medical Center, Chinese PLA General Hospital, Beijing, 100101, China

**Keywords:** Sarcopenia, Frailty, Disability, Creatinine, Cystatin C

## Abstract

**Background:**

The serum creatinine/cystatin C ratio (CCR) and sarcopenia index (SI) are emerging diagnostic markers for sarcopenia, but their values among community-dwelling older adults remain uncertain. This study evaluates the utility of SI and CCR in diagnosing sarcopenia and predicting incident frailty and disability in activities of daily living (ADL) within a substantial cohort of older Chinese adults.

**Methods:**

We conducted a prospective cohort study using data from the baseline survey (2011–2012) and the third wave (2014–2015) of the China Health and Retirement Longitudinal Study (CHARLS). After applying eligibility criteria, 2,574 and 2,357 participants aged ≥60 years were included for analyses of frailty and ADL disability, respectively. Serum creatinine and cystatin C levels were measured to calculate CCR and SI. Receiver operating characteristic (ROC) curves were used to determine cutoff values and evaluate the diagnostic accuracy of these markers for sarcopenia. Multivariate logistic regression models were employed to examine the associations of SI, CCR, and sarcopenia with incident frailty and ADL disability.

**Results:**

Both CCR and SI exhibited significant correlations with age, muscle mass indicators, and handgrip strength. The area under the curve (AUC) for CCR was 0.61 (95% CI: 0.57−0.64) for men and 0.59 (95% CI: 0.56−0.62) for women, while for SI, it was 0.60 (95% CI: 0.56−0.64) for men and 0.63 (95% CI: 0.58−0.67) for women. The difference in AUC between CCR and SI was not statistically significant (P > 0.05). Participants in the highest quartile of SI or CCR had reduced odds of incident frailty (SI: OR = 0.24, 95% CI 0.11−0.52; CCR: OR = 0.24, 95% CI 0.11−0.51) and ADL disability (SI: OR = 0.71, 95% CI 0.54−0.94; CCR: OR = 0.69, 95% CI 0.52−0.91) compared to those in the lowest quartile. Sarcopenia defined by either CCR or SI was independently associated with increased risks of incident frailty (CCR: OR = 1.84, 95% CI: 1.20–2.83; SI: OR = 1.70, 95% CI: 1.12–2.58) and ADL disability after adjusting for confounders.

**Conclusions:**

Both CCR and SI demonstrate weak diagnostic accuracy for sarcopenia, but their performance in predicting frailty and ADL disability was moderate and comparable among community-dwelling older adults. These findings support further investigation of CCR and SI as biomarkers to help clinicians identify older individuals at risk of adverse clinical outcomes.

## 1 Background

Sarcopenia, characterized by the progressive loss of skeletal muscle mass (SMM), strength, and physical performance, is prevalent among older adults and linked to adverse clinical outcomes [[1], [2], [3]] including falls [4], frailty [5], disability [6], and mortality [7]. Preventing sarcopenia is therefore crucial for enhancing the quality of life and overall health of this population [8]. Current diagnostic guidelines rely on device-based measurements of muscle mass, such as computed tomography (CT), dual-energy X-ray absorptiometry (DXA), and bioelectrical impedance analysis (BIA), along with muscle function tests. However, these methods can be time-consuming, expensive, or inaccessible in some settings [9,10]. A simple, cost-effective, and reliable alternative is therefore needed.

Several serum biomarkers have been proposed to aid sarcopenia diagnosis. Serum creatinine (SCr), a byproduct of muscle metabolism, reflects both renal function and muscle mass. However, its accuracy is influenced by diet, hydration, and renal clearance [11]. Cystatin C (CysC), a protein produced by all nucleated cells, is less affected by these factors and provides a more stable estimate of glomerular filtration rate (eGFR). Yet, neither SCr nor CysC alone accurately reflects muscle mass [12].

To address this, two indices combining SCr and CysC have recently been proposed. Kashani et al. [13] introduced a novel index, the serum creatinine/CysC ratio (CCR), which estimates muscle mass, mortality risk, and long-term outcomes in ICU patients. Additionally, Lien et al. developed a sarcopenia index (SI), defined as SCr × CysC-based GFR (eGFR_CysC_) [14], which shows a strong correlation with sarcopenia, potentially stronger than that of the CCR [15].

Despite growing evidence supporting these indices, results remain inconsistent. Some studies suggest that CCR estimates skeletal muscle mass [11,16], while others report modest correlations or low diagnostic performance (e.g., AUCs < 0.7) [[17], [18], [19]]. These discrepancies may reflect differences in populations and muscle assessment methods. Nevertheless, CCR and SI have been associated with clinical outcomes such as malnutrition, frailty, and hospital stay across various settings [13,[20], [21], [22], [23]]. However, a comprehensive evaluation of CCR and SI among Chinese community-dwelling older adults is lacking. Therefore, this study aimed to assess the diagnostic accuracy of CCR and SI for sarcopenia and their ability to predict incident frailty and disability in a large, prospective cohort of older adults in China.

## 2 Methods

### 2.1 Study population

Data were collected from the baseline survey (2011–2012) and the third wave (2014–2015) of the CHARLS, an ongoing cohort study that captures a nationally representative sample of community-dwelling adults aged 45 years or older from 28 provinces in China. The study achieved a response rate of 80.5%, enrolling a total of 17,708 participants. The CHARLS protocol was approved by the ethical review committee at Peking University (approval No. IRB 00001052-11014), and informed consent was obtained from all participants. Further details about the recruitment strategies and study design have been published elsewhere [24].

Serum creatinine and cystatin C levels were measured at baseline. Data on frailty, falls, and disability were collected at both baseline and follow-up. For the present analysis, we included participants aged 60 years and older. Individuals were excluded if they had missing data on key covariates, serum biomarkers, or outcome variables, or if they had chronic kidney disease (eGFR < 60 mL/min/1.73 m²), frailty, or ADL disability at baseline. Two analytic cohorts were established to assess the associations of CCR and SI with adverse outcomes: Cohort 1 (n = 2,574) for incident frailty, and Cohort 2 (n = 2,357) for incident ADL disability.

### 2.2 Assessment of blood biomarkers

Following a standard protocol, venous blood samples were obtained from each participant after an overnight fast by trained personnel from the Chinese Center for Disease Control and Prevention (Chinese CDC). The samples were then promptly transported at 4 °C to local laboratories, where they were separated into plasma and buffy coat and stored in individual cryovials. These cryovials were immediately frozen at −20 °C and transported to the Chinese CDC in Beijing within 2 weeks. Ultimately, the samples were stored at −80 °C until analysis at the laboratory of Capital Medical University. SCr and CysC levels were determined using methods described in the “2011–2012 National Baseline Blood Data Users’ Guide” (http://charls.pku.edu.cn/index/en.html). Specifically, SCr levels were measured using the rate-blanked and compensated Jaffe creatinine method, with within-assay and between-assay coefficients of variation less than 1.60% and 2.10%, respectively. CysC levels were assessed using the particle-enhanced turbimetric assay, with both within-assay and between-assay coefficients of variation less than 5.00%.

The eGFR_CysC_ was calculated based on CysC using the following equation [25]:

eGFR_CysC_ = 86 * CysC^−1.132^.

The CCR was calculated as serum creatinine ÷ CysC, and the SI was calculated as serum creatinine * eGFR_CysC_.

### 2.3 Assessment of sarcopenia status

Sarcopenia status was assessed using the Asian Working Group for Sarcopenia (AWGS) 2019 algorithm, which comprises three components: muscle strength, appendicular skeletal muscle mass (ASM), and physical performance [26].

Sarcopenia is diagnosed when low muscle mass is combined with either low muscle strength or low physical performance. Handgrip strength (kg) was measured in both the dominant and non-dominant hands, with participants exerting maximum force on a Yuejian™ WL-1000 dynamometer (Nantong Yuejian Physical Measurement Instrument Co., Ltd., Nantong, China). The threshold for low grip strength was set at less than 28 kg for men and less than 18 kg for women. ASM was estimated using a validated anthropometric equation designed for Chinese residents [27]. Several studies have confirmed strong agreement between this ASM equation model and dual X-ray absorptiometry (DXA) [28]. The skeletal muscle mass index (SMI) was calculated as ASM divided by height squared, with low muscle mass defined as the lowest 20% of SMI values for each sex within the study population [28,29]. Measurements of body weight and height were conducted using a stadiometer and a digital floor scale, accurate to 0.1 cm and 0.1 kg, respectively [28,29]. Low muscle mass was determined as SMI values less than 4.98 kg/m^2^ in women and less than 6.86 kg/m^2^ in men at baseline in Cohort 1, and less than 5.22 kg/m^2^ in women and less than 6.99 kg/m^2^ in men at baseline in Cohort 2. Physical performance was evaluated by gait speed and the chair stand test, using the method described by Wu et al. [28]. Further details on the definitions for sarcopenia components in the CHARLS have been previously described [28].

### 2.4 Assessment of frailty and disability

Frailty was assessed using a modified version of the Fried's Physical Frailty Phenotype (PFP) [30], which was originally developed for the Cardiovascular Health Study. The assessment included five criteria: slowness, weakness, exhaustion, inactivity, and shrinking. This tool has been adapted and validated for the CHARLS to study the epidemiology and natural progression of frailty among community-dwelling older adults in China [31,32].

Slowness was quantified by measuring the usual gait speed over a 2.5-meter course. Two trials were conducted, and the average walking time was calculated to determine gait speed (m/s). Handgrip strength was assessed using a dynamometer, with two trials for each hand; the highest value from the four trials was recorded. Slowness and weakness were defined based on established cut-offs from prior research in similar populations [30,33]. Exhaustion was evaluated using two items from the Center for Epidemiological Studies-Depression (CES-D) scale [25]: “I felt everything I did was an effort.” And “I could not get going.” Exhaustion was identified if respondents reported feeling this way for “a moderate amount of time; 3–4 days” or “most of the time; 5–7 days” to either question. Physical inactivity was defined as walking less than 10 min continuously during a typical week. Shrinking was determined if an individual reported a weight loss of 5 kg or more in the previous year or had a BMI of 18.5 kg/m² or less, calculated from measured standing height and body weight.

ADL disability was defined as difficulty performing at least one ADL, including dressing, bathing, eating, getting into or out of bed, toileting, and handling urination [34].

### 2.5 Covariates

Sociodemographic characteristics included age (in years), sex, marital status (married vs. unmarried), and educational level (below primary school, primary school and above, and secondary school and above). Lifestyle factors consisted of physical activity, smoking status, and alcohol consumption. Physical activity was defined as belonging to the highest tertile of scores on the International Physical Activity Questionnaire (IPAQ) [35]. Smoking status was categorized into current smokers and non-smokers. Excessive alcohol use was quantified by multiplying the number of days per week alcohol (including liquor, beer, wine, or rice-based beverages) was consumed by the number of drinks (measured in liang, bottles, or mugs) per day for each individual. Consumption of more than 14 drinks per week for men and more than seven drinks per week for women was considered excessive alcohol drinking. Chronic disease status encompassed diagnosed hypertension, diabetes, and heart disease. Hypertension was defined as a systolic blood pressure (BP) of 140 mmHg or higher, diastolic BP of 90 mmHg or higher, a physician’s diagnosis of hypertension, or the use of antihypertensive medication. Diabetes was defined by a self-reported history of diagnosis by a physician, treatment for diabetes, a fasting glucose level (FPG) of 126 mg/dL or higher, or an HbA1c level of 6.5% or higher. The presence of heart disease was identified through a physician diagnosis during an in-person visit with study personnel, as recorded on a questionnaire.

### 2.6 Statistical analysis

Histograms and the Shapiro-Wilk test were used to assess the distribution of continuous variables. The data are presented as means and standard deviations (SDs) for continuous variables and as numbers and percentages for categorical variables. Group differences were analyzed using one-way ANOVA or χ² tests, as appropriate.

The diagnostic values of the CCR and SI for sarcopenia within each cohort were assessed via receiver operating characteristic (ROC) curves for men and women. The optimal CCR and SI cutoff values for men and women were calculated by determining the shortest distance between the ROC curve and the upper left corner of the graph, which maximized the sum of the sensitivity and specificity [36]. The AUCs, sensitivity, specificity, accuracy, positive predictive values, negative predictive values, and corresponding 95% confidence intervals (CIs) were calculated; the larger the AUC, the better the overall diagnostic accuracy [37]. Comparisons between two correlated ROC curves were performed using the DeLong method [38].

Pearson’s correlation analysis was used to determine the relationships of age and serum biomarkers (SI and CCR) with body composition (BMI, SMI, and ASM) and physical function (gait speed and handgrip strength). Univariate and multivariate logistic regression models were applied to calculate the unadjusted and adjusted odds ratios (ORs) and 95% CIs of SI and CCR for incident adverse health outcomes. Both indices were treated as either continuous variable using z-scores (per 1-SD) or categorical variables (using the quartile cutoff points). Associations between CCR-defined and SI-defined sarcopenia for incident frailty in Cohort 1 and incident ADL disability in Cohort 2 were also analyzed using univariate and multivariate logistic regression models, adjusting for the same covariates as above. In addition, we evaluated the predictive performance of CCR-based and SI-based sarcopenia for these outcomes by calculating the AUC and Brier score, which reflects models’ overall discrimination and calibration. Lower Brier scores indicate better calibrated models [39]. All statistical analyses were two-sided, and statistical significance was identified based on a *p*-value of < 0.05. Statistical analysis was performed with Stata (version 17).

## 3 Results

### 3.1 Characteristics of the study population

**Fig. 1:**
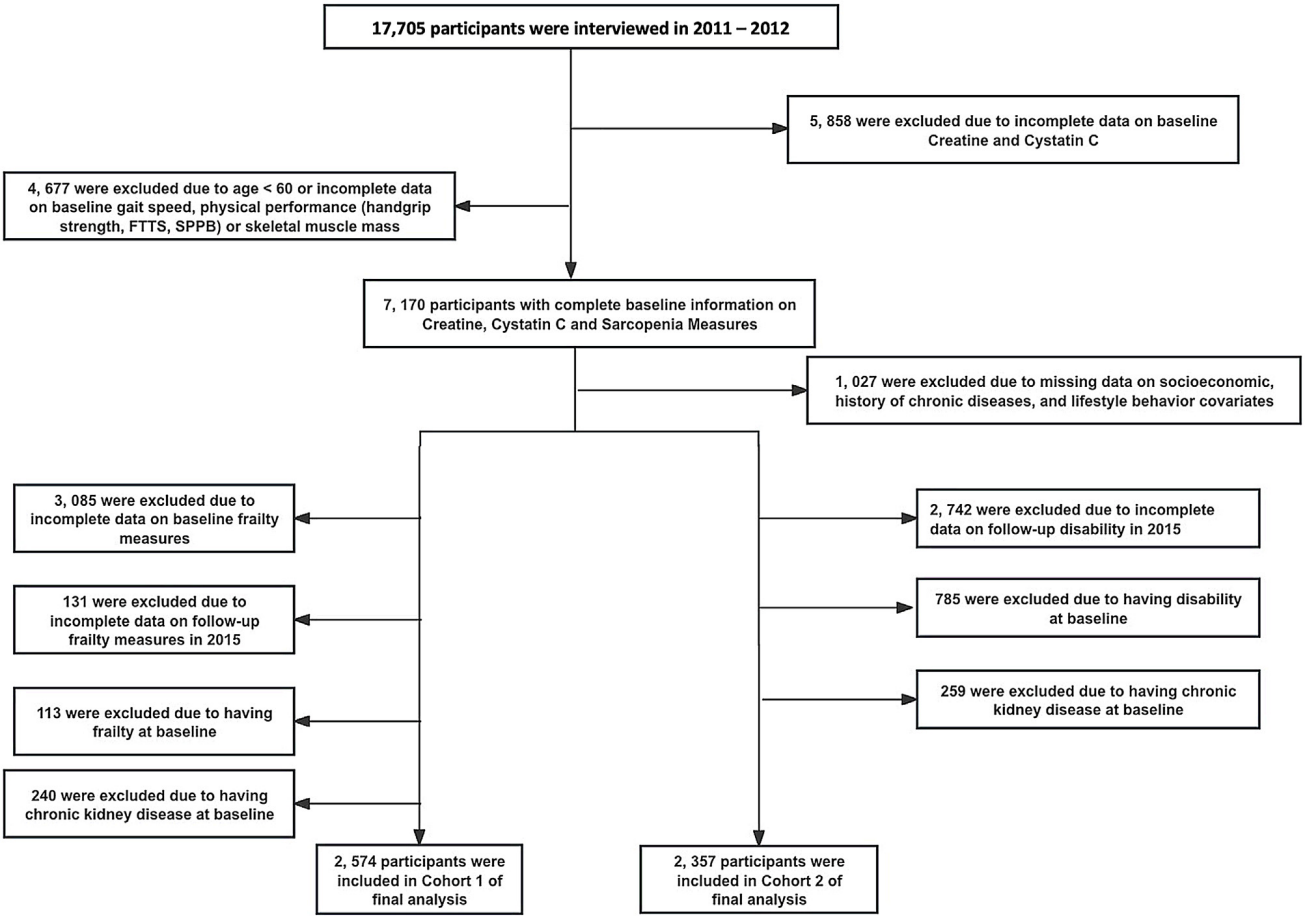
Process for selecting the study population.

A comprehensive analysis involved 2,574 participants in Cohort 1 to investigate incident frailty, while 2,357 participants were examined in Cohort 2 for the assessment of ADL disability. Fig. 1 provides additional details on the study population inclusion process, with nonresponse analysis results comparing included and excluded populations available in Table S1.

The baseline characteristics of the study population, categorized by quartiles of CCR and SI, are presented in Table1 for Cohort 1 (Table S2 for Cohort 2). The mean age of participants in the Q4 group was significantly lower than that of the other three groups. In contrast, individuals in the Q1 group of both CCR and SI tended to be older, with a lower proportion of males, married individuals, excessive drinkers, current smokers, and diagnosed diabetes compared to all other quartiles in both cohorts.

**Table 1:** Baseline characteristics of the study population according to quartiles of serum creatinine and cystatin C-based diagnostic indices in Cohort 1.

		Quartile of Creatinine/Cystatin C ratio					Quartile of Sarcopenia Index				
Characteristic	Total (n = 2, 574)	Q1	Q2	Q3	Q4	P Value	Q1	Q2	Q3	Q4	P Value
Age, years	67.54 ± 6.07	69.03 ± 6.47	67.93 ± 6.08	67.01 ± 5.78	66.18 ± 5.54	<.001	69.21 ± 6.46	67.92 ± 6.05	66.91 ± 5.77	66.11 ± 5.52	<.001
Male, n (%)	1, 144 (44.44)	128 (19.81)	231 (35.98)	351 (54.59)	434 (67.50)	<.001	133 (20.65)	253 (39.35)	338 (52.48)	420 (65.32)	<.001
Married, n (%)	2, 053 (79.76)	456 (70.59)	513 (79.91)	534 (83.05)	550 (85.54)	<.001	453 (70.34)	518 (80.56)	532 (82.61)	550 (85.54)	<.001
Educational Background, n (%)						<.001					
illiterate	1, 523 (59.17)	464 (71.83)	425 (66.20)	355 (55.21)	279 (43.49)	<.001	462 (71.74)	418 (65.01)	359 (55.75)	284 (44.17)	<.001
Primary School or Above	612 (23.78)	123 (19.04)	126 (19.63)	168 (26.13)	195 (30.33)	<.001	122 (18.94)	128 (19.91)	167 (25.93)	195 (30.33)	<.001
Secondary School or Above	439 (17.06)	59 (9.13)	91 (14.17)	120 (18.66)	169 (26.28)	<.001	60 (9.32)	97 (15.09)	118 (18.32)	164 (25.51)	<.001
Physical Activity, n (%)	928 (36.05)	229 (35.45)	235 (36.60)	232 (36.08)	232 (36.08)	0.98	223 (34.63)	246 (38.26)	236 (36.65)	223 (34.68)	0.467
Excessive Drinking, n (%)	355 (13.79)	61 (9.44)	81 (12.62)	97 (15.09)	116 (18.04)	<.001	61 (9.47)	87 (13.53)	92 (14.29)	115 (17.88)	<.001
Present Smoking, n (%)	579 (22.49)	88 (13.62)	134 (20.87)	168 (26.13)	189 (29.39)	<.001	91 (14.13)	145 (22.55)	159 (24.69)	184 (28.62)	<.001
Diagnosed Hypertension, n (%)	1, 315 (51.09)	321 (49.69)	337 (52.49)	317 (49.30)	340 (52.88)	0.447	326 (50.62)	336 (52.26)	315 (48.91)	338 (52.57)	0.534
Diagnosed Diabetes, n (%)	498 (19.35)	103 (15.94)	115 (17.91)	122 (18.97)	158 (24.57)	0.001	101 (15.68)	117 (18.20)	119 (18.48)	161 (25.04)	<.001
Diagnosed Heart Disease, n (%)	412 (16.01)	106 (16.41)	107 (16.67)	95 (14.77)	104 (16.17)	0.795	107 (16.61)	106 (16.49)	94 (14.60)	105 (16.33)	0.732

### 3.2 Distribution of the CCR and SI in the healthy controls and groups with adverse health outcomes

**Fig. 2:**
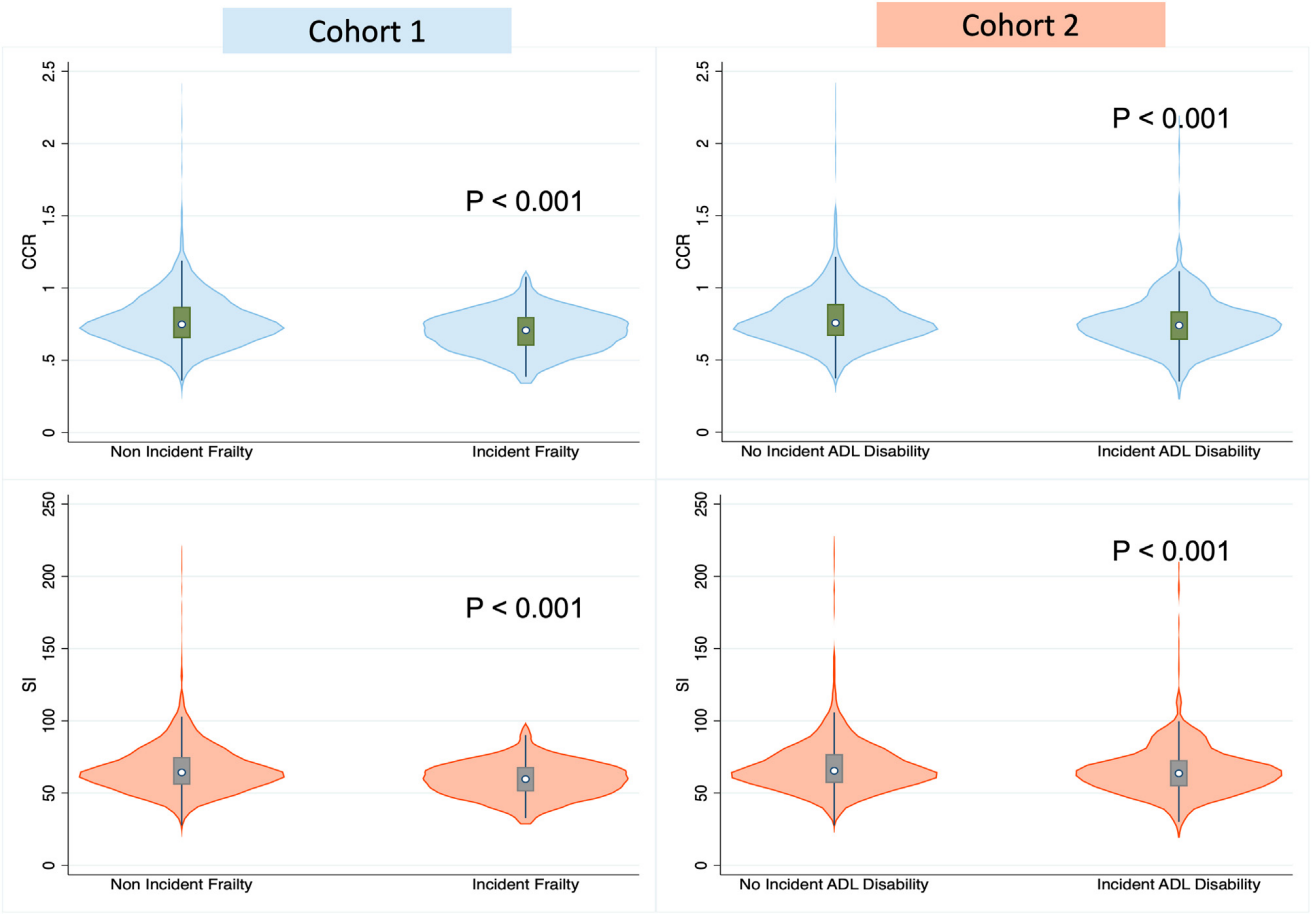
Violin plot and box-plot analysis comparing the distribution of the CCR and SI in the frailty/ADL disability group and healthy controls.

In both cohorts, the CCR and SI exhibited lower values in the frailty/ADL disability group compared with healthy controls, as depicted in Fig. 2.

### 3.3 Correlation between the SI and CCR, age, BMI, SMI, ASM, handgrip strength, and gait speed

**Fig. 3:**
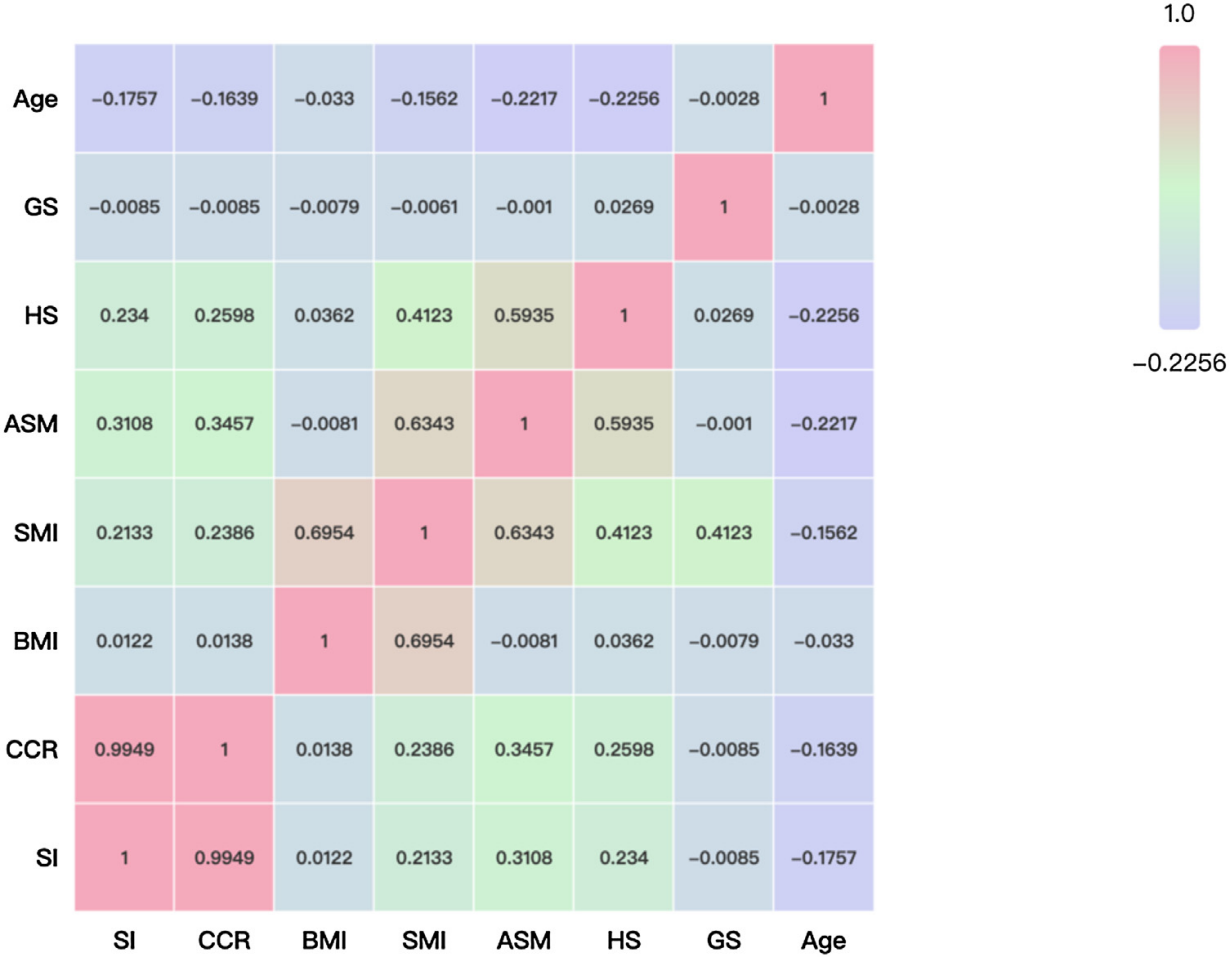
Correlation analysis of serum biomarkers (SI and CCR) with age, body composition (BMI, SMI, ASM), HS and GS in Cohort 1.

Fig. 3 shows the correlation coefficients of CCR, SI, age and BMI with muscle mass and physical function indicators in Cohort 1. Notably, a very strong positive correlation was observed between CCR and SI (r = 0.9949, P < 0.001). The SI showed weak but significant negative correlations with age (r = −0.1757, P < 0.001) and weak positive correlations with SMI (r = 0.2133, P < 0.001), ASM (r = 0.3108, P < 0.001), and handgrip strength (r = 0.2340, P < 0.001). It was not significantly correlated with BMI (r = 0.0122, P = 0.5352) or gait speed (r = -0.0085, P = 0.6650). Similarly, CCR demonstrated weak but significant negative correlations with age (r = −0.1639, P < 0.001) and weak positive correlations with SMI (r = 0.2386, P < 0.001), ASM (r = 0.3457, P < 0.001), and handgrip strength (r = 0.2598, P < 0.001). It was not significantly correlated with BMI (r = 0.0138, P = 0.4854) or gait speed (r = −0.0085, P = 0.6647). The correlation coefficients of biomarkers in Cohort 2 demonstrated similar results (Figure S1).

### 3.4 Associations between the SI, CCR and adverse health outcomes

The effects of SI and CCR on the risk of developing frailty and ADL disability were investigated using logistic regression analyses, as presented in Table 2. In the univariate model, both SI and CCR showed significant associations with the risk of new falls (SI: OR per 1-SD increase 0.99, 95% CI 0.98–0.99; CCR: OR per 1-SD increase 0.32, 95% CI 0.19–0.56) and incident ADL disability (SI: OR per 1-SD increase 0.99, 95% CI 0.99–1.00; CCR: OR per 1-SD increase 0.43, 95% CI 0.26–0.70). These associations persisted even after extensive adjustment for potential confounders, including sociodemographic factors, lifestyle factors, and chronic disease status. In the fully adjusted multivariate model, SI and CCR remained significantly associated with the risk of frailty (SI: OR per 1-SD increase 0.97, 95% CI 0.95–0.99; CCR: OR per 1-SD increase 0.06, 95% CI 0.01–0.27).

**Table 2:** Diagnostic accuracy of CCR and SI for predicting AWGS-defined sarcopenia.

	Cohort 1 (n = 2,574)				Cohort 2 (n = 2,357)			
	Men (n = 1,144)		Women (n = 1,430)		Men (n = 766)		Women (n = 1,591)	
	CCR	SI	CCR	SI	CCR	SI		
Cut-off	0.82	64.82	0.66	56.26	0.83	69.66	0.66	59.82
Sensitivity, %	67.5 (60.7–73.8)	52.2 (45.2–59.1)	53.6 (47.5–59.6)	53.6 (47.5–59.6)	69.7 (60.7–77.7)	67.2 (58.1–75.4)	49.8 (43.6–56.0)	60.5 (54.3–66.4)
Specificity, %	53.6 (50.3–56.8)	67.7 (64.6–70.7)	64.6 (61.8–67.4)	64.6 (61.8–67.4)	55.6 (51.7–59.5)	59.3 (55.4–63.1)	71.8 (69.3–74.2)	62.4 (59.8–65.0)
AUC^a^	0.61 (0.57–0.64)	0.60 (0.56–0.64)	0.59 (0.56–0.62)	0.59 (0.56–0.62)	0.63 (0.58–0.67)	0.63 (0.59–0.68)	0.61 (0.58–0.64)	0.61 (0.58–0.65)
Positive Predictive Value, %	24.5 (21.1–28.3)	26.5 (22.3–31.1)	26.6 (23.0–30.5)	26.6 (23.0–30.5)	22.9 (18.7–27.5)	23.8 (19.4–28.7)	25.9 (22.2–30.0)	24.2 (20.9–27.6)
Negative Predictive Value, %	88.0 (85.1–90.6)	86.4 (83.7–88.8)	85.4 (82.8–87.6)	85.4 (82.8–87.6)	90.6 (87.3–93.3)	90.5 (87.3–93.1)	87.8 (85.8–89.7)	88.9 (86.7–90.8)

CCR = creatinine/cystatin C ratio; SI = sarcopenia index.

a AUC = Area under the receiver operating characteristic curve.

Compared with participants in Q1 of SI, individuals in Q4 of SI exhibited decreased odds of incident frailty (OR 0.24, 95% CI 0.11–0.52, all *P* for trend <0.05) and incident ADL disability (OR 0.71, 95% CI 0.54–0.94) in fully adjusted models. Similarly, participants in Q4 of CCR demonstrated independently reduced risks of incident frailty (OR 0.24, 95% CI 0.11–0.51, all *P* for trend <0.001), new falls (OR 0.65, 95% CI 0.48–0.88, all *P* for trend <0.05), and incident ADL disability (OR 0.69, 95% CI 0.52–0.91) compared to their counterparts in Q1 of CCR.

### 3.5 Diagnostic accuracy of the CCR and SI for sarcopenia

**Fig. 4:**
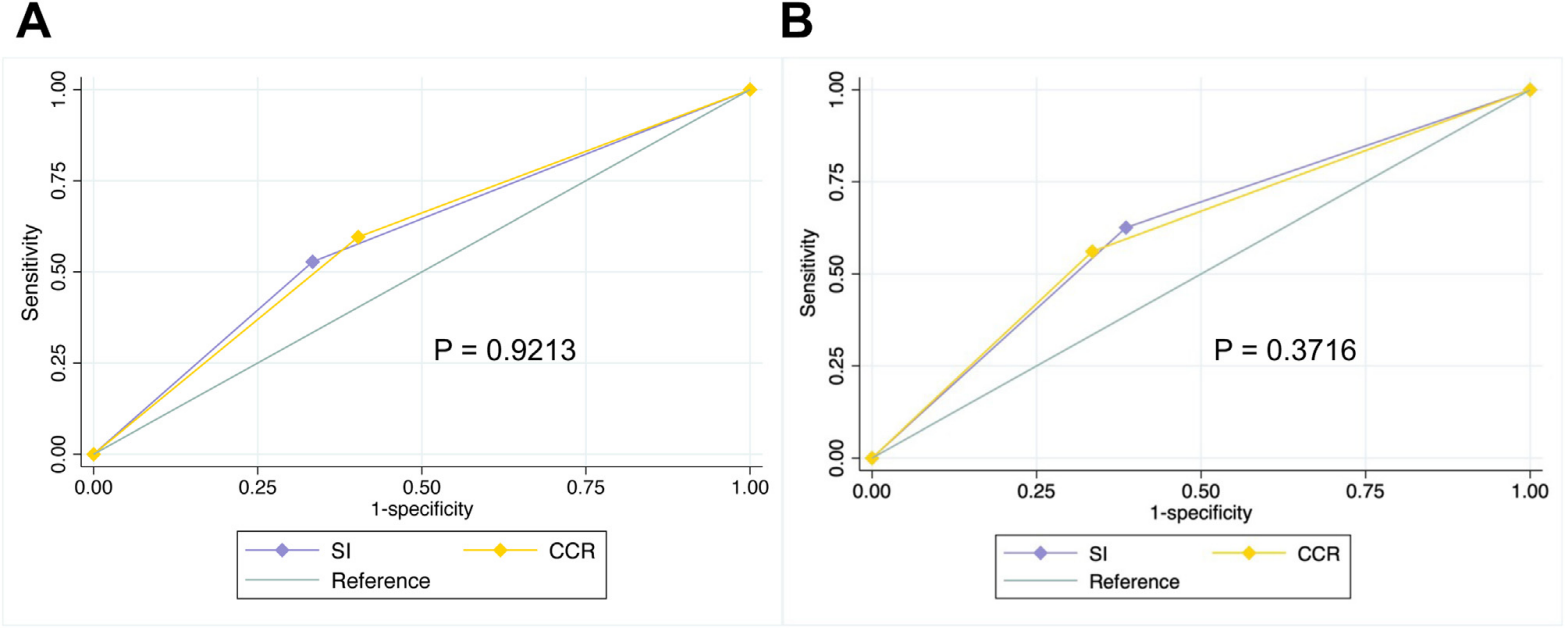
ROC curves of the CCR and SI for diagnosing baseline sarcopenia in A. Cohort 1 and B. Cohort 2.

Fig. 4 shows the results of the ROC analyses for the CCR and SI in two cohorts using AWGS 2019-defined sarcopenia as the reference standard. The optimal CCR cutoff values derived from Cohort 1 were 0.82 for men (sensitivity: 67.5%, specificity: 53.6%) and 0.66 for women (sensitivity: 53.6%, specificity: 64.6%). The optimal SI cutoff values were 64.82 for men (sensitivity: 52.2%, specificity: 67.7%) and 56.26 for women (sensitivity: 53.6%, specificity: 64.6%) (Table 3).

**Table 3:** Univariate and multivariate logistic regression models for incident adverse health outcomes.

Frailty															
	Univariate Analysis			AUC^b^	Brier Score	Multivariate Analysis Model 1			AUC	Brier Score	Multivariate Analysis Model 2			AUC	Brier Score
	OR	95%CI	p-value			OR	95%CI	p-value			OR	95%CI	p-value		
SI (per 1-SD^a^) increase	0.97	0.95–0.98	<.001	0.59	0.04	0.97	0.95–0.98	<.001	0.69	0.04	0.97	0.95–0.99	<.001	0.75	0.04
CCR (per 1-SD) increase	0.06	0.01–0.21	<.001			0.06	0.01−0.25	<.001			0.06	0.01–0.27	<.001		
Quartiles of SI				0.63	0.04				0.69	0.04				0.75	0.04
Q1	Reference														
Q2	0.53	0.31–0.91	0.021			0.54	0.32–0.94	0.029			0.53	0.30–0.92	0.023		
Q3	0.69	0.42–1.13	0.137			0.72	0.43–1.23	0.231			0.74	0.43–1.26	0.264		
Q4	0.21	0.10–0.45	<.001			0.23	0.11–0.50	<.001			0.24	0.11–0.52	<.001		
P for trend	P < .001					P = 0.001					P = 0.001				
Quartiles of CCR				0.62	0.04				0.69	0.04				0.75	0.04
Q1	Reference														
Q2	0.66	0.39–1.09	0.105			0.66	0.39–1.12	0.121			0.65	0.38–1.09	0.104		
Q3	0.63	0.38–1.05	0.078			0.63	0.36–1.10	0.103			0.65	0.37–1.13	0.123		
Q4	0.22	0.11–0.46	<.001			0.23	0.10–0.49	<.001			0.24	0.11–0.51	<.001		
P for trend	P < .001					P < .001					P < .001				

ADL Disability															
	Univariate Analysis			AUC	Brier Score	Multivariate Analysis Model 1			AUC	Brier Score	Multivariate Analysis Model 2			AUC	Brier Score
	OR	95%CI	p-value			OR	95%CI	p-value			OR	95%CI	p-value		
SI (per 1-SD) increase	0.99	0.99–1.00	0.001	0.55	0.20	0.99	0.99–1.00	0.046	0.60	0.20	0.99	0.99–1.00	0.034	0.61	0.20
CCR (per 1-SD) increase	0.43	0.26–0.70	0.001	0.55	0.20	0.6	0.36–0.99	0.045	0.60	0.20	0.57	0.35–0.96	0.033	0.61	0.20
Quartiles of SI				0.56	0.21				0.60	0.20				0.61	0.20
Q1	Reference														
Q2	0.75	0.58−0.96	0.021			0.81	0.63–1.04	0.097			0.82	0.63–1.05	0.121		
Q3	0.89	0.70–1.14	0.362			1.05	0.81–1.35	0.724			1.04	0.81–1.35	0.756		
Q4	0.58	0.45−0.76	<.001			0.72	0.55–0.95	0.019			0.71	0.54–0.94	0.016		
P for trend	P = 0.001					P = 0.116					P = 0.096				
Quartiles of CCR				0.56	0.20				0.60	0.20				0.61	0.20
Q1	Reference														
Q2	0.7	0.55–0.90	0.006			0.75	0.58–0.97	0.028			0.76	0.59–0.98	0.033		
Q3	0.89	0.70–1.14	0.363			1.02	0.79–1.31	0.878			1.01	0.79–1.31	0.917		
Q4	0.58	0.45–0.75	<.001			0.7	0.53–0.92	0.012			0.69	0.52–0.91	0.009		
P for trend	P = 0.001					P = 0.095					P = 0.077				

a SD, standard deviation.

b AUC, area under the curve.

The AUCs of the CCR and SI were 0.61 (95% CI: 0.57–0.64) and 0.60 (95% CI: 0.56–0.64) for men, and 0.59 (95% CI: 0.56–0.62) and 0.63 (95% CI: 0.58–0.67) for women in Cohort 1, respectively. The differences between the CCR and SI AUCs were not statistically significant in both Cohort 1 (*P* = 0.92) and Cohort 2 (*P* = 0.37).

### 3.6 Associations between sarcopenia and adverse health outcomes

In Cohort 1, both CCR-defined and SI-defined sarcopenia were independently associated with an elevated risk of incident frailty (OR = 1.84, 95% CI: 1.20–2.83; and OR = 1.70, 95% CI: 1.12–2.58, respectively) after adjusting for all potential confounders. The AUCs of the fully adjusted models (Model 2) for incident frailty were 0.74 for CCR-defined sarcopenia and 0.73 for SI-defined sarcopenia, indicating moderate discriminative ability. Corresponding Brier scores were 0.04 for both models, suggesting good overall calibration.

Similarly, in Cohort 2, CCR-defined and SI-defined sarcopenia consistently showed higher odds of incident ADL disability across all models. The AUCs in Model 2 were slightly lower (0.61 for both CCR and SI) for incident ADL disability, reflecting modest discrimination. The Brier scores for these models were 0.20, suggesting acceptable calibration despite lower predictive precision. These results support that while both CCR and SI are independently associated with adverse health outcomes, their prognostic performance is modest, especially for ADL disability (Table 4).

**Table 4:** Associations between CCR/SI-defined sarcopenia with incident adverse health outcomes.

	Univariate Analysis					Multivariate Analysis Model 1					Multivariate Analysis Model 2				
	OR	95%CI	p-value	AUC^a^	Brier Score	OR	95%CI	p-value	AUC	Brier Score	OR	95%CI	p-value	AUC	Brier Score
**Frailty**															
CCR-defined sarcopenia	2.2	1.46–3.34	<.001	0.6	0.04	1.88	1.23–2.87	0.004	0.67	0.04	1.84	1.20–2.83	0.005	0.74	0.04
SI-defined sarcopenia	2.1	1.40–3.15	<.001	0.59	0.04	1.76	1.16–2.66	0.007	0.66	0.04	1.7	1.12–2.58	0.013	0.73	0.04
**ADL Disability**															
CCR-defined sarcopenia	1.48	1.24–1.78	<.001	0.55	0.2	1.35	1.12–1.63	0.002	0.6	0.2	1.36	1.12–1.65	0.002	0.61	0.2
SI-defined sarcopenia	1.4	1.17–1.67	<.001	0.54	0.2	1.22	1.01–1.47	0.034	0.6	0.2	1.23	1.02–1.49	0.029	0.61	0.2

a AUC, area under the curve.

## 4 Discussion

The present study suggests that both CCR and SI are inexpensive, objective, and reproducible proxy measures for dynamically estimating muscle mass and identify individuals at risk of sarcopenia. An advantage of using SI and CCR over traditional imaging techniques such as MRI, CT and DXA lies in their lower cost and greater feasibility, especially in community settings. Moreover, these indexes avoid the hazards of radiation, allowing for repeated measurements without concern for radiation injury. We evaluated the utility of CCR and SI in several key domains. First, both CCR and SI were positively correlated with established muscle mass indicators and physical function measures, including SMI, ASM, GS, and HGS, aligning with previous research [17,40,41]. This supports the current sarcopenia definition in guidelines, which assesses sarcopenia by skeletal muscle mass, muscle strength, and physical performance [42]. Second, both CCR and SI emerged as reliable and independent predictors of incident frailty and ADL disability, as confirmed by regression analysis employing these biomarkers in both continuous and categorical forms. Additionally, based on the optimal cutoff values, CCR-defined and SI-defined sarcopenia were significantly associated with incident frailty and ADL disability among Chinese community-dwelling older individuals. These findings underscore the suitability of both CCR and SI as surrogate biomarkers for sarcopenia assessment and prognosis indicators among community-dwelling populations, particularly in predicting adverse clinical outcomes.

Our study revealed a positive correlation among CCR, SI, muscle mass, and handgrip strength, suggesting their potential utility in sarcopenia detection with comparable effectiveness. Previous research has investigated the diagnostic capabilities of SI and CCR, proposing optimal cutoff values for sarcopenia [42]. For instance, one study examining the relationship between CCR and annual chronic obstructive pulmonary disease (COPD) exacerbation rates among COPD patients suggested a CCR cutoff value of 0.71 for predicting AWGS-defined sarcopenia [43]. In another investigation involving Chinese colorectal cancer patients, sex-specific SI cutoff values for predicting sarcopenia were reported as 56.1 in men (sensitivity: 78.3%, specificity: 61.9%) and 43.7 in women (sensitivity: 76.1%, specificity: 68.4%) [44]. These CCR and SI cutoff values varied across studies due to differences in study populations, sarcopenia definitions, and measurement approaches employed to assess sarcopenia subcomponents. Consistent with prior studies [19,45], our findings indicated higher AUC values for SI and CCR in identifying sarcopenia among men compared to women, possibly attributed to men having greater muscle volume. Given that the impact of muscle volume change is less pronounced in CysC than in Cr, it is expected that the decrease in skeletal muscle mass would result in larger changes in CCR and SI among men than women.

Both CCR and SI have demonstrated clinical relevance in specific populations. For example, CCR has been associated with chemotherapy-related adverse effects in lung cancer patients [46], fragility fractures in type 2 diabetes patients [47], and both hospitalizations [48] and mortality among ICU patients [11]. In contrast, the correlation of SI with SMM, sarcopenia, and its predictive capacity for adverse health outcomes has been less explored. Participants with high muscle mass tend to exhibit high SCr levels and low eGFR, whereas those with sarcopenia typically show low creatinine levels and high eGFR. Since eGFRcys offers a more accurate reflection of renal function than eGFRcre, previous studies have established the relationship between sarcopenia and renal function using eGFRcys [49]. Addressing this gap, our study provides evidence that SI can serve as an indicator of muscle mass, HGS, sarcopenia, and several adverse health outcomes with comparable effectiveness to CCR. In a study of 417 colorectal cancer patients, SI exhibited a stronger correlation with body composition than CCR [50]. However, consistent with Tang et al. [51], SI did not demonstrate a stronger association with body composition or physical function, nor did it show significantly higher AUCs in defining sarcopenia compared to CCR in our study.

Our study is the first to investigate the predictive value of SI and CCR for both sarcopenia and adverse health outcomes among community-dwelling older Chinese adults. Previous research includes a single cross-sectional study conducted among Japanese community-dwelling older adults without severe renal function, which presented an effective prediction equation using CCR to estimate SMI [45]. However, this study did not examine the value of SI- and CCR-defined sarcopenia as prognostic indicators for predicting adverse health outcomes among community-dwelling older adults. Skeletal muscle depletion, or sarcopenia, is a common clinical consequence of chronic illness, cancer, aging, poor nutrition, and reduced activity [9,52,53], reflecting prolonged catabolism and decreased functional capacity. Emerging evidence suggests that muscle depletion is a robust prognostic risk factor for postoperative morbidity and mortality. Therefore, the observed associations between SI, CCR, and adverse prognosis may be due to their impact on muscle mass. Although the pathophysiological relationship between muscle loss and prognosis is not fully understood, several studies have suggested associations with a high catabolic state, impaired cytokines, and insulin signaling, leading to glucose intolerance [54]. Sarcopenia also reduces the body’s ability to respond to inflammatory stimuli and delays rehabilitation implementation [54,55]. This reduction in immune function can increase the risk of complications such as recurrent falls, decreased functional ability, prolonged ICU or hospital stays, and ultimately increased frailty risk, as indicated by our findings.

In addition to the observed associations between SI, CCR, and HGS, our study found that both SI- and CCR-defined sarcopenia were independent predictors of incident frailty and disability. Although HGS can be quickly assessed with a dynamometer, it is rarely evaluated in community primary healthcare centers or clinical practice as part of routine medical screening [56]. By contrast, serum creatinine, cystatin C, and albumin are frequently assessed in routine evaluations, making CCR and SI both accessible and reproducible surrogate measures of muscle status. These biomarkers require no specialized equipment and may facilitate the early identification of at-risk individuals in real-world settings. Our results, demonstrating a positive relationship between SI, CCR, and HGS [57,58], are consistent with previous research. Kusunoki et al. [59] reported that CCR was significantly positively correlated with muscle volume and physical function and negatively correlated with body fat mass among community-dwelling elderly people in Japan. Tabara et al. [60] demonstrated that low CCR was independently associated with one-leg standing time, an index of physical performance in older adults. Although there is no direct previous research on the associations between SI, CCR, and SI- and CCR-defined sarcopenia with incident frailty and disability, the associations between sarcopenia and frailty [61,62] and incident disability [63] have been well established. In our analysis, CCR-defined sarcopenia demonstrated slightly higher AUC values than SI-defined sarcopenia in predicting adverse outcomes, suggesting that CCR may have greater prognostic utility in this context. Therefore, rather than proposing CCR and SI as novel diagnostic biomarkers, we recommend them as practical, low-cost, and scalable tools for supporting sarcopenia risk screening and longitudinal monitoring, particularly in settings with limited resources.

To the best of our knowledge, this study is the first to demonstrate the significant association between SI and CCR with incident frailty and ADL disability among Chinese community-dwelling older adults. Additionally, no prior studies have compared the diagnostic accuracy of SI and CCR in detecting sarcopenia within this population. The strengths of this study include its prospective design and population-based setting, with well-established measurements of frailty and sarcopenia. However, several limitations should be acknowledged. First, as an observational study, there is a possibility of bias due to residual confounding, which prevents the establishment of causal links. Nonetheless, our multivariable-adjusted model accounted for various confounding factors, such as diagnosed chronic diseases and physical activity. Second, the assessment of ADL disability relied on self-reported data, which may be subject to recall bias or misclassification, especially among older participants. Third, muscle mass was estimated using validated anthropometric equations rather than gold-standard imaging techniques such as dual-energy X-ray absorptiometry (DXA) or computed tomography (CT), which may introduce measurement error and limit the precision of sarcopenia classification. Future studies should aim to incorporate imaging-based assessments to enhance diagnostic accuracy and comparability across studies. Fourth, the study’s focus on community-dwelling Chinese older adults may limit the generalizability to other populations, particularly younger cohorts. However, given the relative scarcity of research among this population, further investigation into its clinical utility among other ethnicities or younger populations is warranted. Finally, despite these promising findings, the overall diagnostic performance of both indices remains modest, and their utility may vary depending on population characteristics, clinical context, and available resources. Future research should compare these indices with other emerging sarcopenia biomarkers (e.g., myostatin, IL-6), or consider combining them into composite indices. Exploring multimodal approaches that integrate biomarkers with functional assessments may also further improve predictive performance. At present, there is no conclusive evidence favoring one index over the other in clinical practice. We therefore emphasize that these indices are not intended to replace existing diagnostic modalities, such as DXA or bioelectrical impedance analysis (BIA). Instead, they may be more suitable as initial screening tools in resource-constrained settings or as components of a multimodal assessment strategy, particularly where direct measurement of muscle mass is infeasible. We recommend further head-to-head validation of CCR and SI in diverse populations and clinical scenarios to better inform their comparative utility and applicability in practice. Importantly, we also acknowledge that the observed associations between CCR/SI and clinical outcomes such as frailty and ADL disability may reflect alternative underlying mechanisms beyond sarcopenia, and further studies are needed to explore these potential pathways.

## 5 Conclusions

In conclusion, both CCR and SI, based on serum CysC and creatinine, demonstrated modest and remarkably similar diagnostic accuracy for sarcopenia among Chinese community-dwelling older adults. Additionally, both SI and CCR, as well as SI- and CCR-defined sarcopenia, independently predicted incident frailty and ADL disability. These findings suggest that CCR and SI could serve as valuable muscle biomarkers, aiding clinicians in identifying individuals at risk of sarcopenia and its associated adverse health outcomes, thereby facilitating early inclusion in evidence-based primary prevention programs targeting improvements in diet and exercise. Further studies involving larger populations are necessary to validate their utility across diverse ethnic groups.

## CRediT authorship contribution statement

AYB, WHX, JC and BZ contributed to conception and design. AYB and WHX contributed to writing the original draft and reviewing and editing the manuscript. AYB were involved in data curation, and data analysis. JX, JC and BZ contributed to reviewing and editing. WHX, JX and JC contributed to the methodology, project administration, supervision, and verified the underlying data. All authors gave final approval of the version to be published.

## Consent for publication

Not applicable.

## Ethics approval and consent to participate

The CHARLS protocol was approved by Peking University’s ethical review committee (approval No. IRB 00001052-11014), and informed consent was obtained from all participants.

## Funding

This study was supported by Natural Science Foundation of Guangdong Province of China (2023A1515011259).

## Availability of data and materials

Reasonable requests for conditional reuse of the data can be submitted to the corresponding author.

## Declaration of competing interest

None declared.
