# Supplementary material for: Creatinine and cystatin C-based indices for predicting sarcopenia, frailty and disability in older community-dwelling adults
Source: J Nutr Health Aging. 2025 Jul 24;29(9):100635. doi: 10.1016/j.jnha.2025.100635 (PMC12311509; doi:10.1016/j.jnha.2025.100635)
Supplement: Supplementary file 1 [file mmc1.docx]

**Supplementary Material**

**Creatinine and cystatin C-based indices for predicting sarcopenia, frailty and disability in older community-dwelling adults**

**Table S1.** Comparison of the sample dropped and sample used in two cohorts.

**Table S2.** Baseline characteristics of the study population according to quartiles of serum creatinine and cystatin C-based diagnostic indices in Cohort 2

**Figure S1.** Correlation analysis of serum biomarkers (SI and CCR) with age, body composition (BMI, SMI, ASM), HS and GS in Cohort 2.

**Table S1. Comparison of the sample dropped and sample used in two cohorts.**

|  | **Cohort 1** | | | | **Cohort 2** | | | |
| --- | --- | --- | --- | --- | --- | --- | --- | --- |
|  | **Sample Included**  **(N = 2, 574)** | **N** | **Sample Dropped ^a^** | **P Value** | **Sample Included**  **(N = 2, 357)** | **N** | **Sample Dropped** | **P Value** |
| **Characteristic** |  |  |  |  |  |  |  |  |
| **Age, years** | 67.54±6.07 | 2, 272 | 66.97±6.26 | <.001 | 60. 60±9.41 | 2, 531 | 62. 49±10.10 | <.001 |
| **Male, n (%)** | 1, 144 (44.44) | 2, 272 | 1, 263 (55.59) | <.001 | 766 (32.50) | 2, 531 | 1, 233 (48.72) | <.001 |
| **Married, n (%)** | 2, 053 (79.76) | 2, 270 | 1, 829 (80.50) | 0.518 | 1, 998 (84.77) | 2, 531 | 2, 119 (83.72) | 0.316 |
| **Educational Background, n (%)** |  | 2, 270 |  | 0.003 |  | 2, 529 |  | 0.172 |
| **illiterate** | 1, 523 (59.17) |  | 1, 246 (54.89) |  | 1, 323 (56.13) |  | 1, 352 (53.46) |  |
| **Primary School or Above** | 612 (23.78) |  | 640 (28.19) |  | 522 (22.15) |  | 593 (23.45) |  |
| **Secondary School or Above** | 439 (17.06) |  | 353 (15.55) |  | 512 (21.72) |  | 584 (23.09) |  |
| **Physical Activity, n (%)** | 928 (36.05) | 2, 270 | 748 (32.95) |  | 872 (37.00) | 2, 530 | 730 (28.85) | 0.847 |
| **Excessive Drinking, n (%)** | 355 (13.79) | 2, 272 | 394 (17.34) | <.001 | 224 (9.50) | 2. 531 | 383 (15.13) | <.001 |
| **Present Smoking, n (%)** | 579 (22.49) | 1, 778 | 427 (24.02) | 0.242 | 439 (18.63) | 2, 177 | 473 (21.73) | <.001 |
| **Diagnosed Hypertension, n (%)** | 1, 315 (51.09) | 2, 270 | 1, 102 (48.55) | 0.077 | 1, 062 (45.06) | 2, 528 | 1, 119 (44.26) | 0.577 |
| **Diagnosed Diabetes, n (%)** | 498 (19.35) | 2, 265 | 280 (12.36) | <.001 | 418 (17.73) | 2, 530 | 467 (18.46) | 0.511 |
| **Diagnosed Heart Disease, n (%)** | 412 (16.01) | 2, 244 | 295 (13.15) | 0.005 | 358 (15.19) | 2, 498 | 340 (13.61) | 0.117 |

*Notes: The “dropped sample” refers to participants who were excluded from the final analytic cohorts due to missing data on key covariates, serum biomarkers, or outcomes (in Cohort 1 as frailty and Cohort 2 as ADL Disability). We hope this revision improves the clarity of the supplementary table.*

**Table S2. Baseline characteristics of the study population according to quartiles of serum creatinine and cystatin C-based diagnostic indices in Cohort 2**

|  |  | **Quartile of Creatinine/Cystatin C ratio** | | | |  | **Quartile of Sarcopenia Index** | | | |  |
| --- | --- | --- | --- | --- | --- | --- | --- | --- | --- | --- | --- |
| **Characteristic** | **Total**  **(n = 2, 574)** | **Q1** | **Q2** | **Q3** | **Q4** | **P Value** | **Q1** | **Q2** | **Q3** | **Q4** | **P Value** |
| **Age, years** | 60. 60±9.41 | 62.89±10.00 | 61.02±9.40 | 59.76±9.15 | 58.71±8.54 | <.001 | 63.34±10.04 | 61.23±9.32 | 59.37±9.05 | 58.46±8.43 | <.001 |
| **Male, n (%)** | 766 (32.50) | 89 (15.08) | 131 (22.24) | 216 (36.67) | 330 (56.03) | <.001 | 96 (16.27) | 148 (25.13) | 209 (35.48) | 313 (53.14) | <.001 |
| **Married, n (%)** | 1, 998 (84.77) | 454 (76.59) | 510 (86.59) | 507 (86.08) | 527 (89.47） | <.001 | 451 (76.44) | 506 (85.91) | 513 (87.10) | 528 (89.64) | <.001 |
| **Educational Background, n (%)** | |  |  |  |  | <.001 |  |  |  |  | <.001 |
| **illiterate** | 1, 323 (56.13) | 388 (65.76) | 351 (59.59) | 327 (55.52) | 257 (43.63) | | 389 (65.93) | 346 (58.74) | 324 (55.01) | 264 (44.82) | <.001 |
| **Primary School or Above** | 522 (22.15) | 112 (18.98) | 118 (20.03) | 128 (21.73) | 164 (27.84) | | 110 (18.64) | 118 (20.03) | 135 (22.92) | 159 (26.99) | <.001 |
| **Secondary School or Above** | 512 (21.72) | 90 (15.25) | 120 (20.37) | 134 (22.75) | 168 (28.52) | | 91 (15.42) | 125 (21.22) | 130 (22.07) | 166 (28.18) | <.001 |
| **Physical Activity, n (%)** | 872 (37.00) | 214 (36.27) | 214 (36.33) | 227 (38.54) | 217 (36.84) | 0.746 | 214 (36.27) | 213 (36.16) | 229 (38.88) | 216 (36.67) | 0.837 |
| **Excessive Drinking, n (%)** | 224 (9.50) | 37 (6.27) | 45 (7.64) | 51 (8.66) | 91 (15.45) | <.001 | 37 (6.27) | 49 (8.32) | 49 (8.32) | 89 (15.11) | <.001 |
| **Present Smoking, n (%)** | 439 (18.63) | 77 (13.05) | 85 (14.43) | 114 (19.35) | 163 (27.67) | <.001 | 80 (13.56) | 92 (15.62) | 111 (18.85) | 156 (26.49) | <.001 |
| **Diagnosed Hypertension, n (%)** | 1, 062 (45.06) | 274 (46.44) | 260 (44.14) | 258 (43.80) | 270 (45.84) | 0.202 | 283 (47.97) | 267 (45.33) | 246 (41.77) | 266 (45.16) | 0.759 |
| **Diagnosed Diabetes, n (%)** | 418 (17.73) | 88 (14.92) | 98 (16.64) | 104 (17.66) | 128 (21.73) | 0.008 | 89 (15.08) | 92 (15.62) | 108 (18.34) | 129 (21.90) | 0.017 |
| **Diagnosed Heart Disease, n (%)** | 358 (15.19) | 96 (16.27) | 90 (15.28) | 80 (13.58) | 92 (15.62) | 0.617 | 99 (16.78) | 89 (15.11) | 83 (14.09) | 87 (14.77) | 0.614 |

**Figure S1.** Correlation analysis of serum biomarkers (SI and CCR) with age, body composition (BMI, SMI, ASM), HS and GS in Cohort 2.


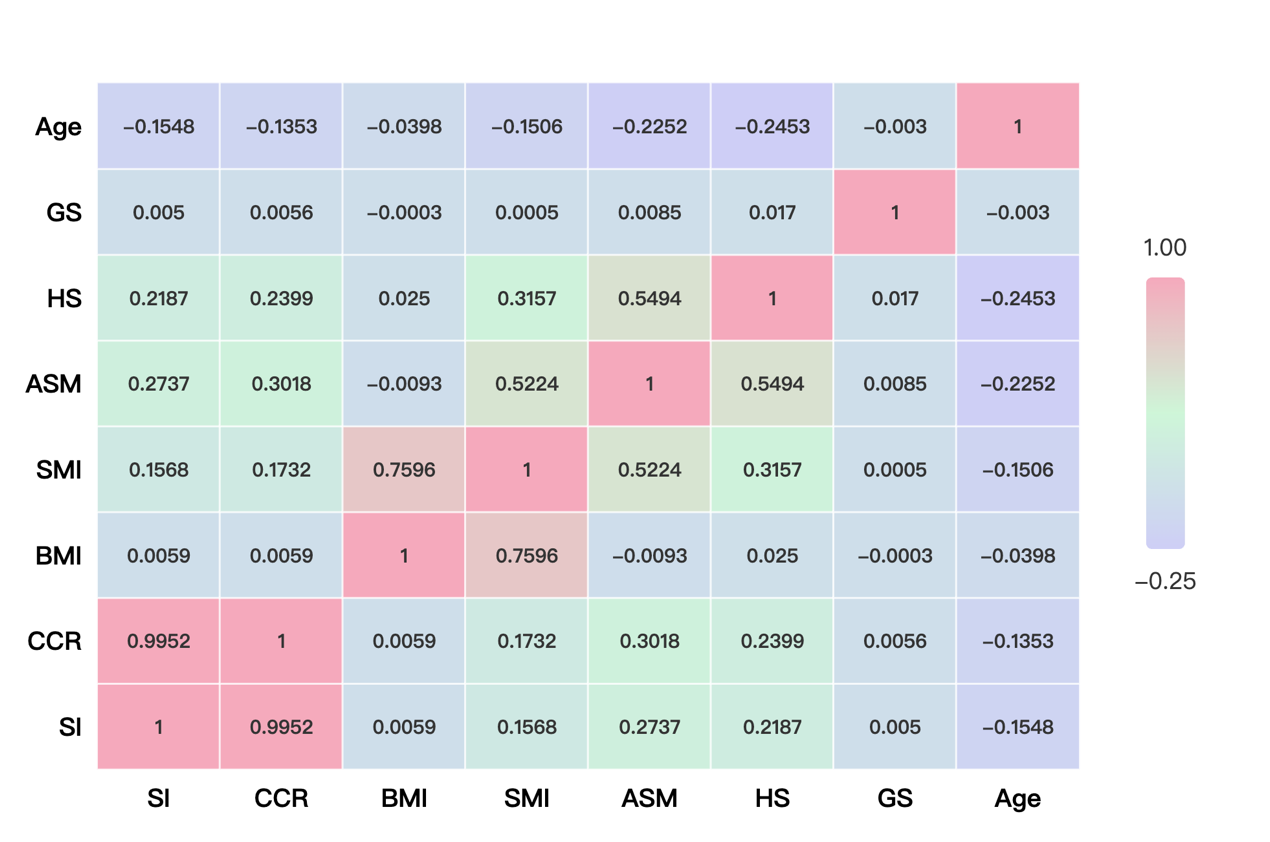


*Notes: BMI, body mass index; SMI, skeletal muscle index; ASM, appendicular skeletal muscle mass; HS, handgrip strength; GS, gait speed.*
